# Gene family information facilitates variant interpretation and identification of disease-associated genes in neurodevelopmental disorders

**DOI:** 10.1186/s13073-020-00725-6

**Published:** 2020-03-17

**Authors:** Dennis Lal, Patrick May, Eduardo Perez-Palma, Kaitlin E. Samocha, Jack A. Kosmicki, Elise B. Robinson, Rikke S. Møller, Roland Krause, Peter Nürnberg, Sarah Weckhuysen, Peter De Jonghe, Renzo Guerrini, Lisa M. Niestroj, Juliana Du, Carla Marini, EuroEPINOMICS-RES Consortium, Rudi Balling, Nina Barisic, Stéphanie Baulac, Hande Caglayan, Dana C. Craiu, Peter De Jonghe, Christel Depienne, Renzo Guerrini, Ingo Helbig, Helle Hjalgrim, Dorota Hoffman-Zacharska, Johanna Jähn, Karl M. Klein, Bobby P. C. Koeleman, Vladimir Komarek, Roland Krause, Eric Leguern, Anna-Elina Lehesjoki, Johannes R. Lemke, Holger Lerche, Taria Linnankivi, Carla Marini, Patrick May, Hiltrud Muhle, Deb K. Pal, Aarno Palotie, Felix Rosenow, Susanne Schubert-Bast, Kaia Selmer, Jose M. Serratosa, Ulrich Stephani, Katalin Štěrbová, Pasquale Striano, Arvid Suls, Tina Talvik, Sarah von Spiczak, Yvonne G. Weber, Sarah Weckhuysen, Federico Zara, James S. Ware, Mitja Kurki, Padhraig Gormley, Sha Tang, Sitao Wu, Saskia Biskup, Annapurna Poduri, Bernd A. Neubauer, Bobby P. C. Koeleman, Katherine L. Helbig, Yvonne G. Weber, Ingo Helbig, Amit R. Majithia, Aarno Palotie, Mark J. Daly

**Affiliations:** 1 Epilepsy Center, Neurological Institute, Cleveland Clinic, Cleveland, OH USA; 2 Stanley Center for Psychiatric Research, The Broad Institute of Harvard and M.I.T, Cambridge, MA USA; 3 Analytic and Translational Genetics Unit, Massachusetts General Hospital, Boston, USA; 4 Cologne Center for Genomics, University of Cologne, Cologne, Germany; 5 Genomic Medicine Institute, Lerner Research Institute Cleveland Clinic, 9500 Euclid Avenue, Cleveland, OH 44195 USA; 6 Luxembourg Centre for Systems Biomedicine, University of Luxembourg, 6, Avenue du Swing, 4367 Belvaux, Luxembourg; 7 Wellcome Sanger Institute, Wellcome Genome Campus, Hinxton, UK; 8 Department of Epidemiology, Harvard T.H. Chan School of Public Health, Boston, MA USA; 9 The Danish Epilepsy Centre, Dianalund, Denmark; 10 Institute for Regional Health research, University of Southern Denmark, Odense, Denmark; 11 Center for Molecular Medicine Cologne, University of Cologne, Cologne, Germany; 12 Cologne Excellence Cluster on Cellular Stress Responses in Aging-Associated Diseases, University of Cologne, Cologne, Germany; 13 Division of Neurology, Antwerp University Hospital, Antwerp, Belgium; 14 Neurogenetics Group, Center for Molecular Neurology, VIB, Antwerp, Belgium; 15 Laboratory of Neurogenetics, Institute Born-Bunge, University of Antwerp, Antwerp, Belgium; 16 Pediatric Neurology and Neuroscience Department, Children’s Hospital Anna Meyer, University of Florence, Florence, Italy; 17 National Heart & Lung Institute and MRC London Institute of Medical Science, Imperial College London, London, UK; 18 Division of Clinical Genomics, Ambry Genetics, Aliso Viejo, CA USA; 19 CeGat and Practice for Human Genetics, Tübingen, Germany; 20 Epilepsy Genetics Program, Boston Children’s Hospital, Boston, MA USA; 21 Department of Neuropediatrics UKGM, University of Giessen, Giessen, Germany; 22 Department of Genetics, University Medical Center Utrecht, Utrecht, The Netherlands; 23 Division of Neurology, Children’s Hospital of Philadelphia, Philadelphia, PA USA; 24 Department of Neurology and Epileptology, Hertie Institute for Clinical Brain Research, University of Tübingen, Tübingen, Germany; 25 Department of Epileptology and Neurology, University of Aachen, Aachen, Germany; 26 The Epilepsy NeuroGenetics Initiative (ENGIN), Children’s Hospital of Philadelphia, Philadelphia, PA USA; 27 Department of Biomedical and Health Informatics, Children’s Hospital of Philadelphia, Philadelphia, PA USA; 28 Department of Neurology, University of Pennsylvania, Perelman School of Medicine, Philadelphia, PA 19104 USA; 29 Division of Endocrinology, Department of Medicine, University of California, San Diego, CA USA; 30 Institute for Molecular Medicine Finland, University of Helsinki, Helsinki, Finland

**Keywords:** Paralogs, Gene family, Conservation, Missense variants, Neurodevelopmental disorders

## Abstract

**Background:**

Classifying pathogenicity of missense variants represents a major challenge in clinical practice during the diagnoses of rare and genetic heterogeneous neurodevelopmental disorders (NDDs). While orthologous gene conservation is commonly employed in variant annotation, approximately 80% of known disease-associated genes belong to gene families. The use of gene family information for disease gene discovery and variant interpretation has not yet been investigated on a genome-wide scale. We empirically evaluate whether paralog-conserved or non-conserved sites in human gene families are important in NDDs.

**Methods:**

Gene family information was collected from Ensembl. Paralog-conserved sites were defined based on paralog sequence alignments; 10,068 NDD patients and 2078 controls were statistically evaluated for de novo variant burden in gene families.

**Results:**

We demonstrate that disease-associated missense variants are enriched at paralog-conserved sites across all disease groups and inheritance models tested. We developed a gene family de novo enrichment framework that identified 43 exome-wide enriched gene families including 98 de novo variant carrying genes in NDD patients of which 28 represent novel candidate genes for NDD which are brain expressed and under evolutionary constraint.

**Conclusion:**

This study represents the first method to incorporate gene family information into a statistical framework to interpret variant data for NDDs and to discover new NDD-associated genes.

## Background

**Fig. 1:**
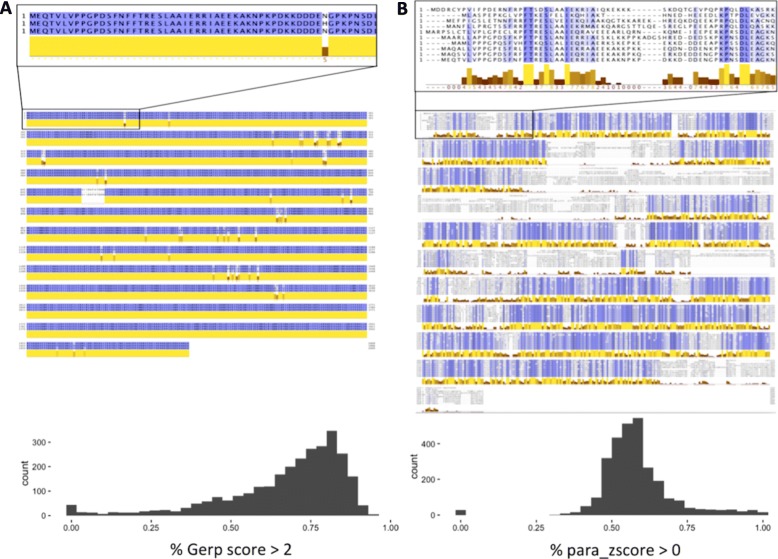
Vertical (ortholog) vs. horizontal (paralog) conservation. Top: protein sequence alignment of voltage-gated sodium channels. Top left: alignment of *Homo sapiens* (NP_001159435.1), *Bos taurus* (NP_001180147.1), and *Mus musculus* (NP_001300926.1) SCN1A protein sequences. High sequence similarity is depicted by violet amino acid coloring and yellow conservation bars below the alignment using JalView. Top right: protein alignment in JalView of all members of the human voltage-gated sodium channel gene family (*SCN1A*, *SCN2A*, *SCN3A*, *SCN4A*, *SCN5A*, *SCN7A*, *SCN8A*, *SCN9A*, *SCN10A*, *SCN11A*). This alignment of paralogs shows less conservation compared to the alignment of *SCN1A* to its vertical cross-species orthologs on the left. Bottom left: GERP score analysis over all genes within gene families (homolog conservation is measured by the percentage of all nucleotides per gene with GERP scores > 2). Bottom right: distribution percentage of nucleotides per gene within gene families having para_zscores > 0. Conservation between close homologs is generally much more uniform and homogeneous than conservation between paralogs

Differentiating risk-conferring from benign missense variants represents a major challenge in clinical practice to diagnose rare and genetic heterogeneous neurodevelopmental disorders (NDDs). Protein sequence conservation is one of the main underlying assumptions for methods evaluating the pathogenicity of missense variants. It is commonly determined by aligning mammalian or vertebrate protein sequences to identify conserved sites among orthologs. The high average sequence similarity of homologs of disease-associated genes often translates into highly conserved sequence profiles (Fig. 1a). Recent large-scale sequencing studies on NDDs have independently identified multiple paralogous genes associated with the same or related NDD (e.g., the family of voltage-gated sodium channel genes: *SCN1A*, *SCN2A*, *SCN8A*; the family of chromodomain helicase DNA-binding proteins: *CHD2*, *CHD4*, *CHD8*) [1–4]*.* This observation raises the question whether other genes within the same gene family are also associated with NDDs. Since in the aforementioned NDD sequencing studies truncating variants in paralogs often show consistent associations to NDDs, we sought to explore whether paralog information could refine our interpretation of missense variation. Paralogs often have similar protein sequences (Fig. 1b), and amino acids conserved across all paralogs might well be critical for protein function. As such, variants changing paralog-conserved residues may plausibly be more deleterious than variants changing residues in paralog non-conserved sites and therefore be more likely to confer risk to disease. Two previous studies have highlighted the utility of systematic functional annotation of disease-causing residues across human paralogs for genes associated with long QT syndrome, Brugada syndrome, and catecholaminergic polymorphic ventricular tachycardia [5, 6]. Both studies showed improved variant interpretation by comparing corresponding mutations in paralogs in patients with the same phenotype.

Statistical power for the discovery of disease-associated genes is the greatest in genetically homogeneous patient groups. NDDs are phenotypically and genetically heterogeneous. Several of the NDD disease-associated genes are pleiotropic and appear in clinically distinct NDDs (sub-NDDs) indicating a shared molecular pathology. Even in NDD cohorts with > 1000 trios, the majority of disease-associated genes have fewer than 10 de novo variants [1–4]. To increase the statistical power, gene set enrichment analysis is often applied to discover pathways associated with diseases of the same or a similar phenotype [5, 6]. The 3348 human protein-coding gene families accounted for 72% of the protein-coding genes. It has been shown before that approximately 80% of disease-associated genes have paralogs in human [7]. To our knowledge, it has not been empirically investigated on a genome-wide scale whether disease-associated missense variants reside in paralog-conserved or non-conserved sites. In this manuscript, we evaluate the use of “paralog conservation” and provide evidence that this information offers a powerful addition to variant annotation and disease gene discovering in NDDs.

## Methods

### Patient and genetic data

We analyzed exome sequencing variant data from 10,068 neurodevelopmental disorder (NDD) trios (probands and their unaffected parents) including 3982 autism (ASD), 5226 developmental delay (DD), and 822 severe epilepsy (EPI) patients. The ASD cohort was derived from published studies [2, 8]. The DD cohort combined previously published de novo DD and ID studies which used similar cohort inclusion/exclusion criteria [3, 4, 9–11]. The EPI cohort included published trio data sets from (356/822 trios, 43%) [1, 12] as well as 466 (56% of the 822 trios) exome-wide de novo data that were recently published [13]. As control data, we used variant data from 2078 trios sequenced with the same technology as the ASD patient cohort. These controls are unaffected siblings of the ASD patients [2, 10].

To ensure uniformity in the variant representation and annotation across published datasets and with respect to the ExAC reference database [14], we created a standardized variant representation using a Python implementation of vt [15] and re-annotated all variants from the different datasets with ANNOVAR [16] using the RefSeq and Ensembl gene annotations (2016Feb01) and the conservation score GERP++ [17].

### Definition of gene family and paralog conservation

Ensembl defines gene families based on maximum likelihood phylogenetic gene trees [18] using the longest translated protein annotated in CCDS [19] for each gene. Paralogs are then defined as genes of the same species related by a duplication event (as an inner tree node). First, we downloaded the human paralog definitions using the Ensembl BioMart system [20] representing each gene with an Ensembl gene identifier. The paralogs could be grouped into 3584 gene families. Ensembl IDs were then converted to HGNC gene names. Non-coding genes and genes without a HGNC symbol were excluded, and only gene families with at least two HGNC genes were used for further analysis. CCDS data were downloaded the same day as HGNC and Ensembl data (v20150512).

In total, 3348 gene families were defined for 13,382 HGNC genes; 1815 families contained three or more paralogs. We extracted the longest transcript from CCDS for each HGNC gene and constructed for each gene family a multiple sequence alignment with MUSCLE [21] including all paralog protein sequences. Evolutionary younger paralogs show higher functional redundancy [22]. To avoid alignments of strongly diverging sequences and to increase overall similarity, we built sub-groups for each gene family using pairwise alignment length cutoffs of > 80% aligned residues [23]. Clusters (sub-families) were defined by connected components within a protein family alignment similarity graph in which two genes with > 80% aligned residues were connected through an edge. Only clusters with at least two proteins were further processed. In total, we generated 2871 (sub) gene families comprising 8233 genes. Each sub-group was re-aligned using MUSCLE. The MUSCLE output was then processed as input for JalView [24] to generate conservation scores for each alignment position. The conservation score calculation in JalView is based on the AMAS method of multiple sequence alignment analysis [25]. Conservation is measured here as a numerical index reflecting the conservation of physico-chemical properties in the alignment. Amino acid identity scores the highest, and substitutions to amino acids lying in the same physico-chemical class scored higher than from different classes. For each HGNC CCDS gene, the conservation scores at each position were extracted from the JalView. Finally, to identify amino acids of high and low paralog conservation and to make scores comparable between genes, the mean and the standard deviation conservation score over all amino acids per gene were calculated to compute a paralog conservation *z*-score (para_zscore) per amino acid position by subtracting the mean from the original score dividing the difference by the standard deviation. Residues with positive para_zscores are defined as paralog conserved and residues with negative values as paralog non-conserved (Additional file 1: Figure S1A and Figure S2).

### Comparison of paralog and ortholog conservation

To compare paralog and ortholog conservation, we collected for every gene having human paralogs all orthologous protein sequences down to vertebrates using ENSEMBL BioMart and processed the ortholog sequences analogous to the paralog workflow described above. We generated multiple sequence alignments using MUSCLE, calculated conservation for each position of the alignment using JalView using the AMAS scoring, and calculated a *z*-score for the orthologous conservation analogous to the paralog conservation for each residue of the gene. By definition, as for the paralogs, conserved means *z*-score > 0 and non-conserved *z*-score ≤ 0. Then, we compared for each gene how many and which positions were conserved in orthologous or/and paralogous proteins. The similarity between paralog and ortholog conservations was calculated using the Rand Index (RI) [26] using the *rrand* function of the *phyclust* 0.1-28 R package (available from https://CRAN.R-project.org/package=phyclust) and Pearson’s correlation with the *cor.test* method. All calculations were done in R version 3.41.

### Gene family enrichment analysis

To identify gene families with significant mutational burden, we adopted a de novo expectation model [27] to assess mutation rates for nonsense, frameshift, or canonical splice disruptions (collectively termed protein-truncating variants (PTVs)) and missense variants for gene families (missense+PTV). We derived gene-based rates of de novo mutations from the local gene sequence context and summed the expectations and the observed counts for all genes within each gene family [14]. The expected and observed numbers of de novo mutations in each variant class for NDD combined were compared using a Poisson distribution. Notably, the discovery of de novo burden in a gene family is more challenging compared to the single gene analysis because of larger amount of expected mutations due to combining the expectations from all gene family members (including those which are not expressed in the tissue of interest, e.g., brain). We used a Bonferroni-corrected significance threshold of 0.05 for the 2871 gene families tested. Furthermore, to exclude “passenger” variants and enrich for true disease variants, we excluded de novo variants also seen in adult individuals without early onset NDDs in ExAC (*n* = 60,706 exomes) prior to the enrichment analysis. Variants absent from this population reference panel, which is a proxy for standing variation in the human population, are more likely to be deleterious. This very stringent filter reduces the number of false-positive disease-causing variants.

### Enrichment analysis of paralog-conserved vs. non-conserved sites

Similar to the gene family enrichment analysis above, we adopted the de novo expectation model [27] to assess the mutation rates for missense variants only. In this site-specific enrichment analysis, we considered only missense variants and missense expectations for each gene family. We classified every amino acid position within each gene into “conserved sites” with paralog conservation (para_zscore > 0, residues with higher paralog conservation than the gene-specific mean) and “non-conserved sites” without paralog conservation (para_zscore ≤ 0) and summed the observations for both groups independently across the family. The expected missense mutation rates were adjusted for the size of the paralog-conserved sites and non-conserved sites. The observed variant counts were assigned to either of these two groups depending on the paralog conservation state of the mutated amino acid residue for all members within each gene family (Additional file 1: Figure S1B). The expected and observed numbers of de novo mutations in each variant class for NDDs combined were compared using a Poisson distribution. To exclude “passenger” variants and enrich for disease variants, we excluded de novo variants present as standing variation in the 60,706 individuals in ExAC prior to the enrichment analysis.

### Identification of brain-expressed genes and evolutionary constraint

We extracted brain expression data from the Genotype-Tissue Expression (GTEx) consortia [28] data and considered genes with > 1 read per kilobase of transcript per million mapped reads (RPKM) in brain tissues as “brain-expressed.” Gene loss-of-function intolerance (pLI) scores and gene missense intolerance scores were derived from ExAC. We considered genes with missense *z*-scores > 3.09 or pLI scores ≥ 0.9 as intolerant of variants. Genes were classified as plausible novel disease genes for NDD if they were present in an exome-wide enriched gene family, brain expressed, and under constraint (either missense or PTV).

## Results

### Paralog conservation of missense de novo mutations in NDD patients

**Fig. 2:**
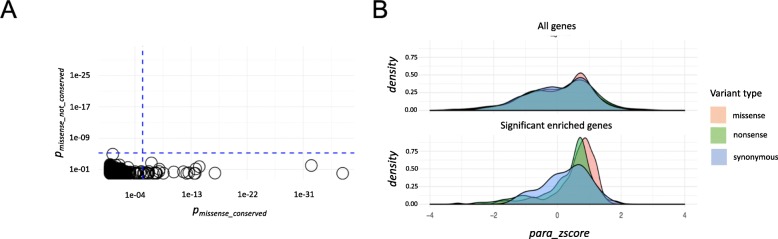
Assessment of paralog conservation. **a** Identification of missense variant gene family enrichment in NDD patients for paralog-conserved missense variants. NDD-associated missense variants are enriched in paralog-conserved sites. *y*-axis: missense variant enrichment analysis considering only paralog non-conserved sites across genes of each gene family (para_zcore ≤ 0, *p*_missense_not_conserved_). *x*-axis: missense variant enrichment analysis considering only paralog-conserved sites (para_zcore > 0, *p*_missense_conserved_). None of the gene families shows exome-wide significant enrichment for paralog non-conserved sites. Twenty-six gene families (depicted by circles) show exome-wide significant de novo missense variant burden at paralog-conserved sites. The significance threshold was calculated by Bonferroni correction for testing 5 × 2871 gene families (*P* = 3.48 × 10^−6^) and is depicted by the blue dotted line. **b** Enrichment of missense variants in paralog-conserved sites in genes with significant DNM burden in this study. Distribution of NDD patient missense, nonsense, and synonymous para_zscores for all non-significantly enriched genes (top) and genes significantly enriched for DNM missense variants (bottom panel) depicted by density plots. DNM burden was calculated using the mutational framework described by Samotcha et al. (for details, see the “Methods” section). Genes were categorized into two groups: those with a significant burden and those without. In disease-associated genes (those with DNM burden), missense variants were enriched at paralog-conserved sites relative to missense variants in non-significantly enriched genes (*P* value < 2.2E−16, top vs. bottom panel). Missense variants in genes without DNM burden were not enriched at paralog-conserved sites compared to synonymous variants (*P* value = 0.1157, top panel). In genes with DNM burden (bottom panel), missense variants were significantly enriched at paralog-conserved sites compared to synonymous variants (*P* value = 3.01 × 10^−4^). The same test for nonsense variants vs. synonymous variants did not show significant differences in paralog conservation (*P* value = 0.3913). *P* values were calculated using a Wilcoxon test

To investigate the degree to which de novo mutations (DNMs) in NDDs are enriched in paralog-conserved sites, we compared the variant distribution of DNMs in 10,068 NDD patients to 2078 individuals without NDDs. To increase the signal, we excluded those DNMs present in ExAC [29]. We evaluated the contribution of paralog-conserved and non-conserved missense variants to NDDs. Our analysis included 2871 gene families encapsulating 8233 genes. We observed 27 significantly enriched (*P* < 3.48 × 10^−6^) gene families in the patient cohort that were only identified in an analysis of paralog-conserved missense variants, but none in the parallel, comparably powered, analysis of non-conserved sites only (Fig. 2a). Although many of these genes also show a burden for protein-truncating variants (PTVs), the paralog enrichment is specific for missense variants since we did not identify a shift towards paralog-conserved sites for nonsense variants (Fig. 2b). This is in line with the nonsense-mediated decay as the expected disease mechanism for PTV mutations, regardless of the variant position within the protein sequence. Furthermore, missense variant enrichment at paralog-conserved sites is not detectable in genes without a DNM burden in this study.

### Gene family enrichment in NDDs and sub-phenotypes

After establishing that disease-associated missense variants are enriched at paralog-conserved sites (Additional file 1: Figure S1), we assessed the degree to which gene family information could assist in discovering novel disease-associated genes using the cohort of 10,068 patients with NDDs. We extended the approach of Samocha et al. [27] (see the “Methods” section) to gene families to identify gene families with significant enrichment of mutations in NDD patients. We included any protein-truncating variants (PTVs) across the entire sequence as well as all missense variants at paralog-conserved protein sites absent from ExAC into this analysis.

**Fig. 3:**
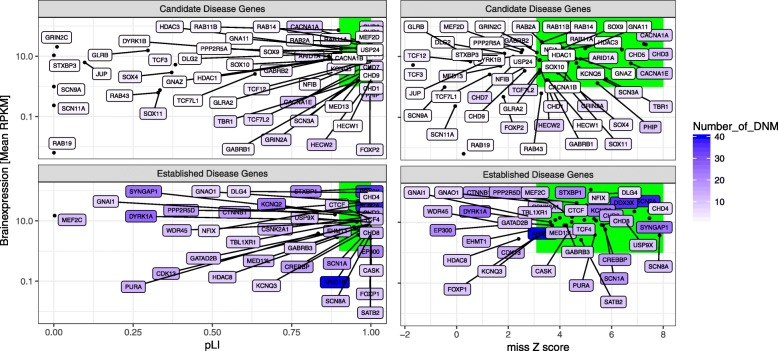
Established NDD disease genes are brain expressed and under evolutionary constraint. Every dot represents a gene of the 43 DNM enriched gene families. The colors of the box and font represent the number of DNMs (N.DNM) identified in the gene in 10,668 NDD trios. *y*-axis: brain gene expression level in RPKM derived from the GTEx expression dataset; *x*-axis: gene constraint scores (left: pLI, indicating gene LoF intolerance; right: missense *z*-score, indicating gene missense intolerance). Disease-associated DNMs are likely to affect brain-expressed and evolutionary constrained genes (defined as brain expression RPKM > 1, constraint score pLI ≥ 0.9 and missense *z*-score > 3.09; green boxes). In support of this hypothesis, we observe that all previously known and frequently mutated genes are brain expressed and under evolutionary constraint

We identified 43 gene families (1.49% of all gene families) enriched for de novo paralog-conserved missense and PTVs (Bonferroni correction significance threshold for testing 5 × 2871 gene families = 3.48 × 10^−6^; Table 1). In all 43 gene families, the most frequently mutated gene and often additional genes harboring de novo variants are brain-expressed (Fig. 3). Within the enriched gene families, 94 paralogs carried at least one DNM vs. 59 paralogs without any DNM. In total, 7.47% of all NDD patients carried a de novo paralog-conserved missense or PTV in the 43 enriched gene families. In the NDD patients, we found 753 DNMs in 43 gene families while only 49.92 DNMs were expected (*P* ≪ 1.0 × 10^−100^). The paralog-conserved missense variant enrichment signal of these genes was 7.8-fold (observed DNMs, 261; expected DNMs, 33.01). There was no enrichment if we examined the paralog non-conserved missense variants in this group of genes (observed DNMs, 41; expected DNMs, 31.03). No enrichment was observed in the 2078 individuals without a NDD (observed DNMs, 5; expected DNMs, 10.34, *P* = 1.0). The majority of the frequently mutated genes have previously been established as disease-associated genes by demonstrating an exome-wide significant DNM burden in disease-specific single gene enrichment studies (Table 1, highlighted in black and bold) [1–4, 10]. When removing all the established disease genes from the analysis, we still observe a 4.72-fold enrichment (observed, 162 DNMs in the 43 enriched genes families; expected, 34.27, *P* = 6.10 × 10^−56^). This enrichment increases to 5.28-fold when we removed all non-brain-expressed genes from the 43 enriched genes families (28.71 DNM expected vs. 149 DM observed, *P* = 3.72 × 10^−57^).

**Table 1:** Forty-three significantly enriched gene families in the combined de novo paralog-conserved missense and PTV analysis for 10,068 NDD trios. Only enriched gene families significant after applying the Bonferroni significance threshold for testing 5 × 2871 gene families (3.48 × 10^−6^) are included. Gene names highlighted in red are affected by DNM and the number of DNM is indicated inside the soft brackets. Genes in bold have not previously been reported as significantly enriched in exome-wide ASD, DD, or EPI studies

Gene families	DNMs expected	DNMs observed	*P* value	5264 DD patients	3982 ASD patients	822 EPI patients	2087 controls
*ARID1B (40)*, *ARID1A (5)*	**0.96**	**45**	**4.28E−58**	**39**	**6**	**0**	**0**
*SCN2A (38)*, *SCN1A (18)*, *SCN8A (11)*, *SCN3A (4)*, *SCN11A (2)*, *SCN9A (1)*, ***SCN5A***, ***SCN7A***, ***SCN4A***, ***SCN10A***	**6.16**	**74**	**1.77E−52**	**36**	**16**	**22**	**0**
*DDX3X (35)*, ***DDX3Y***	**0.49**	**35**	**8.94E−52**	**34**	**1**	**0**	**0**
*DYRK1A (26)*, *DYRK1B (1)*	**0.37**	**27**	**1.89E−40**	**21**	**5**	**1**	**0**
*EP300 (20)*, *CREBBP (16)*	**1.39**	**36**	**8.81E−38**	**30**	**5**	**1**	**1**
*KCNQ2 (23)*, *KCNQ3 (7)*, *KCNQ5 (3)*	**1.14**	**33**	**3.09E−36**	**27**	**2**	**4**	**0**
*SYNGAP1 (24)*, ***DAB2IP***, ***RASAL2***	**1.33**	**24**	**4.35E−22**	**17**	**6**	**1**	**0**
*STXBP1 (20)*, ***STXBP3 (1)***, ***STXBP2***	**0.88**	**21**	**5.93E−22**	**14**	**2**	**5**	**0**
*GRIN2B (16)*, *GRIN2A (6)*, *GRIN2C (1)*, ***GRIN2D***	**1.89**	**23**	**1.46E−17**	**17**	**3**	**3**	**0**
*CTNNB1 (16)*, *JUP (1)*	**0.79**	**17**	**2.27E−17**	**15**	**2**	**0**	**0**
*CHD2 (16)*, *CHD1 (3)*	**1.15**	**19**	**4.00E−17**	**10**	**8**	**1**	**0**
*PURA (13)*, ***PURB***	**0.41**	**13**	**1.13E−15**	**12**	**0**	**1**	**0**
*CHD3 (10)*, *CHD4 (6)*, *CHD5 (5)*	**1.94**	**21**	**3.45E−15**	**19**	**2**	**0**	**0**
*TCF4 (11)*, *TCF12 (3)*, *TCF3 (2)*	**0.93**	**16**	**5.76E−15**	**14**	**2**	**0**	**1**
*CDK13 (14)*, ***CDK12***	**0.66**	**14**	**1.71E−14**	**13**	**1**	**0**	**0**
*PPP2R5D (16)*, *PPP2R5A (1)*, ***PPP2R5B***, ***PPP2R5C***, ***PPP2R5E***	**1.25**	**17**	**3.62E−14**	**16**	**1**	**0**	**1**
*WDR45 (11)*, ***WDR45B***	**0.32**	**11**	**7.60E−14**	**9**	**0**	**2**	**0**
*MEF2C (11)*, *MEF2D (2)*, ***MEF2A***	**0.58**	**13**	**7.77E−14**	**9**	**2**	**2**	**0**
*EHMT1 (13)*, ***EHMT2***	**0.66**	**13**	**3.93E−13**	**13**	**0**	**0**	**0**
*FOXP1 (10)*, *FOXP2 (4)*, ***FOXP4***	**0.86**	**14**	**6.02E−13**	**12**	**2**	**0**	**0**
*FOXG1 (11)*, ***FOXQ1***, ***FOXN3***, ***FOXN2***	**0.39**	**11**	**6.36E−13**	**7**	**1**	**3**	**0**
*CHD8 (12)*, *CHD7 (7)*, *CHD9 (1)*	**2.30**	**20**	**7.73E−13**	**11**	**9**	**0**	**0**
*CSNK2A1 (12)*, ***CSNK2A3***, ***CSNK2A2***	**0.63**	**12**	**5.00E−12**	**10**	**1**	**1**	**0**
*CACNA1E (10)*, *CACNA1A (9)*, *CACNA1B (1)*	**2.72**	**20**	**1.53E−11**	**13**	**1**	**6**	**0**
*HDAC8 (9)*, *HDAC3 (2)*, *HDAC1 (1)*, *HDAC2*	**0.83**	**12**	**1.06E−10**	**11**	**0**	**1**	**0**
*GNAO1 (7)*, *GNAI1 (7)*, *GNAZ (1)*, *GNA11 (1)*, ***GNA14***, ***GNAI3***, ***GNAT2***, ***GNAT3***, ***GNAT1***, ***GNAI2***, ***GNA15***, ***GNAQ***	**1.82**	**16**	**1.31E−10**	**13**	**2**	**1**	**0**
*CASK (8)*, *DLG4 (6)*, *DLG2 (1)*, ***DLG1***, ***DLG3***	**1.58**	**15**	**1.67E−10**	**12**	**1**	**2**	**0**
*GATAD2B (10)*, ***GATAD2A***	**0.65**	**10**	**2.10E−09**	**10**	**0**	**0**	**0**
*MED13L (12)*, *MED13 (2)*	**1.68**	**14**	**3.47E−09**	**10**	**4**	**0**	**0**
*TBL1XR1 (9)*, ***TBL1Y***, ***TBL1X***	**0.52**	**9**	**5.01E−09**	**7**	**2**	**0**	**0**
*PHIP (10)*, *BRWD3 (2)*	**1.46**	**13**	**5.64E−09**	**10**	**2**	**1**	**0**
*CTCF (9)*, ***CTCFL***	**0.56**	**9**	**9.00E−09**	**7**	**2**	**0**	**0**
*RAB11A (3)*, *RAB2A (2)*, *RAB11B (2)*, *RAB14 (2)*, *RAB19 (1)*, *RAB43 (1)*, ***RAB39A***, ***RAB25***, ***RAB4B***, ***RAB39B***, ***RAB4A***, ***RAB30***, ***RAB2B***	**1.01**	**11**	**1.14E−08**	**9**	**2**	**0**	**0**
*GABRB3 (8)*, *GABRB2 (3)*, *GLRA2 (1)*, *GABRB1 (2)*, *GLRB (1)*, ***GLRA1***, ***GABRR1***, ***GABRD***, ***GABRR3***, ***GABRP***, ***GLRA4***, ***GABRQ***, ***GABRR2***, ***GLRA3***	**2.35**	**15**	**3.15E−08**	**7**	**4**	**4**	**0**
*TCF20 (9)*, *RAI1 (3)*	1.40	12	3.33E−08	11	1	0	1
*NFIX (6)*, *NFIA (2)*, *NFIB (2)*, ***NFIC***	0.90	10	4.31E−08	8	2	0	1
*USP9X (10)*, *USP24 (1)*, ***USP9Y***	1.18	11	5.29E−08	9	2	0	0
*SATB2 (9)*, ***SATB1***	0.75	9	1.04E−07	9	0	0	0
*HECW2 (9)*, *HECW1 (1)*	1.04	10	1.61E−07	8	1	1	0
*SOX11 (4)*, *SOX4 (3)*, *SOX9 (1)*, *SOX10 (1)*, ***SOX17***, ***SOX1***, ***SOX8***, ***SOX7***	0.82	9	2.21E−07	9	0	0	0
*ZBTB18 (6)*, ***ZBTB3***	0.28	6	5.43E−07	6	0	0	0
*TCF7L2 (6)*, *TCF7L1 (1)*	0.53	7	1.48E−06	4	3	0	0
*TBR1 (6)*, ***EOMES***	0.35	6	2.01E−06	1	5	0	0

**Fig. 4:**
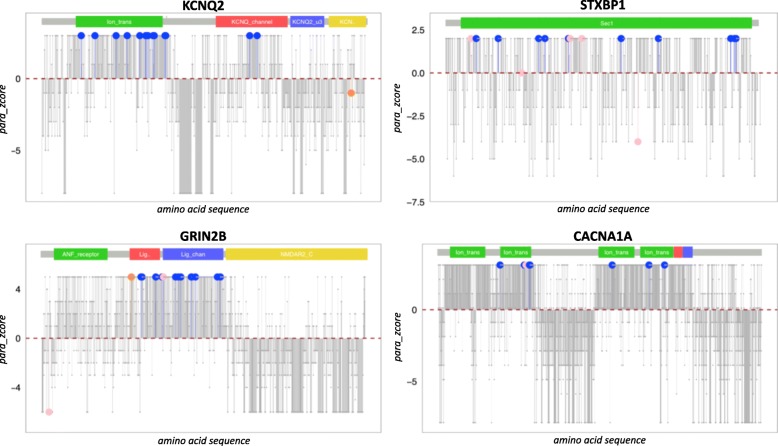
Visualization of para_zscores for *KCNQ2*, *STXBP1*, *CACNA1A*, and *GRIN2B*. Protein sequence is plotted from left to right. Each bar and dot represent one amino acid. Amino acids affected by a missense mutation in the NDD cohort are colored blue, patient PTVs are depicted in pink, and synonymous variants in orange. Amino acid residues with no mutations are colored gray. *y*-axis: para_zscore. Positive values indicate paralog conservation, and the highest score indicates that these amino acids are identical over all gene family members. The red dotted lines indicate the mean paralog conservation of each protein sequence, and the bars below the mean indicate regions of low paralog conservation, thus higher sequence variability over all members of the gene family

Several of these genes, previously not associated with any disease, represent likely NDD-associated genes based on the gene expression, single patient, and mouse gene knockout studies (Additional file 1: Table S1). In one out of the 43 enriched gene families, we observe that the novel NDD-associated gene harbors more DNM variants than the established disease-associated gene of the same gene family (*CHD3* vs. *CHD4*; Table 1). Four gene families show a genome-wide gene family enrichment without prior evidence for any gene on the genome-wide level, even though some individual genes are known disease genes (Additional file 1: Table S1). These gene families include the *RAB2A/B-RAB4A/B-RAB11A/B-RAB14-RAB19-RAB25-RAB30-RAB39A/B-RAB43*-, the *HECW1/2*-, the *SOX1/4/7/8/9/10/11*-, and the *TCF7L1/2*- family. To find further evidence of disease association for less frequently mutated gene family members, we systematically investigated the evolutionary variant intolerance (constraint) [27] and brain expression levels for all mutated paralog genes within the enriched gene families (Fig. 3). We observed that 28 paralog genes of the enriched gene families with DNMs are under evolutionary constraint and brain expressed (Additional file 1: Table S1), showing the same signature as the known disease genes in the same families (Fig. 4). Although none of these genes has previously been reported to be significant on an exome-wide level, 60.71% (17/28) of the novel disease-associated genes have been previously reported in the literature in patients carrying a rare single nucleotide or copy number variant affecting the gene (Additional file 1: Table S1). For 64.28% (18/28) of the genes, available mouse models show neurological and/or behavioral phenotypes supporting the disease association. Given these multiple lines of evidence, in addition to the sequence and expression pattern similarity to the known disease genes in the same families, we consider this list of 28 genes as highly promising candidate disease genes.

### Paralog vs. ortholog conservation

In total, we analyzed 4,869,838 residue positions in the genes within paralog families; 564,758 (12%) were only paralog and 411,284 (8%) only ortholog conserved; 3,393,797 (80%) positions were either conserved or not-conserved in both. Using the adjusted Rand Index (RI) as a similarity measure to compare to equal (number of residue positions) binary vectors for paralog and ortholog conservation, the adjusted RI was 0.3590 (RI = 1 would be identical), clearly showing that both conservation measures are capturing different residues of proteins while having a large overlap. Using Pearson’s correlation method, the correlation between paralog and ortholog conservation was 0.6 (*P* value < 2.2E−16, 95% CI [0.5994, 0.6008]). This shows that paralog- and ortholog-conserved sites are moderately correlated but do not show a strong correlation.

## Discussion

Across multiple analyses in this study, we empirically demonstrate that disease-associated missense variants are enriched at paralog-conserved sites and that this information can offer substantial value in mutation annotation on top of the widely used annotation methods. We developed a gene family DNM enrichment framework and computed a novel amino acid paralog conservation metric, applicable to 42% of all genes in the human genome. Application of the genome-wide paralog conservation metric demonstrates that pathogenic variants affect paralog-conserved sites in NDDs. In contrast, non-conserved sites are more frequently mutated in our tested controls, for which the vast majority of variants are presumed benign. It has recently been proposed that conserved residues within gene family members (paralogs) are under evolutionary constraint and that in silico annotations of known disease-associated residues across families of related proteins can guide variant interpretation [30]. Our results support the idea that paralogs share a similar “core” molecular function of the ancestral gene, since variants in these sites are enriched for patient missense variants indicating hereby a reduction in evolutionary fitness. Evolutionary younger paralogs show higher functional redundancy [22]. To control the functional diversity within gene families and to increase the within-family sequence similarity, we built gene family sub-groups for each defined gene family using pairwise alignment length cutoffs of > 80% aligned amino acids [23].

Sequence conservation across gene families has been extensively discussed in the literature. On the one hand, orthologous domain pairs tend to be significantly more structurally similar than paralogous pairs at the same level of sequence identity [31]. On the other hand, it has been shown that paralogs are functionally not necessarily redundant, and the average fitness cost of loss of a paralogous gene is at least equal deleting single non-paralogous genes in yeast [32]. In addition, protein complexes have been documented to use paralog switching as a mechanism for the regulation of complex stoichiometry [33]. For example, many receptors in the human brain consist of multiple protein subunits, many of which have multiple paralogs and are differentially expressed across brain regions and developmental stages. The brain can tune the electrophysiological properties of synapses to regulate plasticity and information processing by switching from one protein variant to another [34]. Such condition-dependent variant switch during development has been demonstrated in several neurotransmitter systems including NMDA and GABA [33]. Naturally, the question arises whether paralog-conserved or non-conserved sites of the protein sequence are essential for function.

NDDs represent a genetic and phenotypic heterogeneous group of diseases for which pathogenic variants in individual disease genes are rare. Using a gene family version of a recently established DNM enrichment framework [27] for 10,068 NDD patients, we identified 43 PTV and paralog-conserved missense DNM-enriched gene families. Besides highlighting four gene families with genome-wide gene family enrichment without carrying any previously exome-wide established disease gene, the *RAB2A/*B-*RAB4A/B*-*RAB11A/B*-*RAB14*-*RAB19*-*RAB25*-*RAB30*-*RAB39A/B*-*RAB43*, the *HECW1/2*, the *SOX1/4/7/8/9/10/11*, and the *TCF7L1/2*, we additionally report 28 genes showing for the first time statistical support as disease genes. Pathogenic variants in all of these genes are too rare to reach individual gene-wise exome-wide significant enrichment. However, the individual genes belong to gene families that show exome-wide significant enrichment, are brain expressed, and are under evolutionary constraint in the general population [14]. Notably, three of the 43 enriched gene families belong to chromatin helicase DNA-binding protein gene families. Besides the established NDD genes, we observe also five novel candidate disease genes in this group (*CHD1*, *CHD3*, *CHD5*, *CHD7*, *CHD9*) with DNMs in 20 patients in this study. All five genes represent valid candidates for the association of NDDs based on our detailed analysis (Additional file 1: Table S1). Chromatin remodeling is one of the mechanisms by which gene expression is regulated developmentally [35], perhaps explaining the susceptibility to NDDs when mutated. Additionally, four of the brain-expressed, constrained genes that we identified through the new paralog conservation test and presented in the first pre-print version of the manuscript [36], *CHD3*, *CACNA1E*, *PHIP*, and *GABRB2*, were recently shown to be significantly enriched in NDDs with epilepsy [13].

The vast majority of the current variant interpretation methods are scoring single nucleotide variants, and the resulting amino acid changes but do not give a score for every amino acid position. The discrimination from pathogenic to benign variants of these scores can be similar to paralog conservation scoring (Additional file 1: Figure S3); however, only the minority of scores can shed light on functional essential protein regions. Paralog conservation can be used to identify stretches of (paralog) conserved residues. These stretches can overlap functional domains; however, not all annotated domains are paralog conserved and harbor disease variants (Fig. 4 and Additional file 1: Figure S4). Thus, visualization of paralog conservation over the entire protein sequence represents a new, biologically interpretable method for variant classification that is able to highlight and discriminate functional important sites based on the conservation with the gene family. We propose that plotting the paralog conservation is a useful tool to highlight likely functional important protein regions showing high paralog conservation, and thus intuitively supports variant prioritization (e.g., for functional testing or drug target development). Paralogs share similar protein sequences or structural features, e.g., similar binding pockets, e.g., a given compound may show an increased affinity to bind members of the same gene family, possibly resulting in unexpected cross-reactivity and undesired side effects. Usage of the paralog conservation metric in drug target design could therefore have the potential in ruling out or to reduce such cross-reactivity effects. Paralog conservation plots are available on the PER [37] webpage (http://per.broadinstitute.org).

Although our results demonstrate the utility of paralog conservation, the ideal composition of the gene family and choice of protein isoform may differ depending on the individual research question. In addition, while conservation across orthologs or paralogs can be indicative of the necessary function of a given domain, the absence of conservation does not a priori exclude functionally important domains within a protein. This consideration may be particularly relevant for diseases with a later onset which are less under evolutionary selection.

## Conclusions

Overall, we provide empirical evidence using published de novo variants from more than 10k NDD cases that disease-associated missense variants are enriched at paralog-conserved sites. We demonstrate that integration of paralog conservation can be leveraged as a powerful method for variant interpretation and discovery of new NDD disease-associated genes. We provide a pre-computed genome-wide paralog conservation annotation file for all human paralogs as individual files. This resource should enable data and molecular scientists to classify and visualize variants, genes, and proteins of interest and to integrate paralog conservation with existing variant annotation tools.

## Supplementary information

**Additional file 1:** Supplementary methods, figures, and table.

## Data Availability

The code for generating the underlying alignments and the para_zscore, additional code, and examples as well as the paralog conservation metric per gene is available under https://git-r3lab.uni.lu/genomeanalysis/paralogs. Additional data and annotations for the hg19 and hg38 missense variants are available from Zenodo: 10.5281/zenodo.802847 [38]. Source code for generating paralog scores and using them together with ANNOVAR [16] are available from https://git-r3lab.uni.lu/genomeanalysis/paralogs [39]. Paralog conservation plots are available on the PER [37] webpage (http://per.broadinstitute.org). Variant data used in this study were collected from the used studies [2–4, 8–11, 13]. GTEx [28] data was downloaded from https://gtexportal.org/home/.

## Abbreviations

ASD: Autism spectrum disorder
DD: Developmental delay
DNM: De novo mutation
EPI: Epilepsy
GABA: Gamma-aminobutyric acid
HGNC: HUGO Gene Nomenclature Committee
NDD: Neurodevelopmental disorder
NMDA: *N*-methyl-d-aspartate
pLI: Gene loss-of-function intolerance
PTV: Protein-truncating variant
RI: Rand Index
RPKM: Read per kilobase of transcript per million mapped reads

## References

[1] Allen AS, Berkovic SF, Cossette P, Delanty N, Dlugos D, Eichler EE (2013). De novo mutations in epileptic encephalopathies. Nature..

[2] De Rubeis S, He X, Goldberg AP, Poultney CS, Samocha K, Ercument Cicek A (2014). Synaptic, transcriptional and chromatin genes disrupted in autism. Nature..

[3] Fitzgerald TW, Gerety SS, Jones WD, van Kogelenberg M, King DA, McRae J (2014). Large-scale discovery of novel genetic causes of developmental disorders. Nature..

[4] McRae JF, Clayton S, Fitzgerald TW, Kaplanis J, Prigmore E, Rajan D (2017). Prevalence and architecture of de novo mutations in developmental disorders. Nature..

[5] Fromer M, Pocklington AJ, Kavanagh DH, Williams HJ, Dwyer S, Gormley P (2014). De novo mutations in schizophrenia implicate synaptic networks. Nature..

[6] Homsy J, Zaidi S, Shen Y, Ware JS, Samocha KE, Karczewski KJ (2015). De novo mutations in congenital heart disease with neurodevelopmental and other congenital anomalies. Science..

[7] Dickerson JE, Robertson DL (2012). On the origins of Mendelian disease genes in man: the impact of gene duplication. Mol Biol Evol.

[8] Iossifov I, O’Roak BJ, Sanders SJ, Ronemus M, Krumm N, Levy D (2014). The contribution of de novo coding mutations to autism spectrum disorder. Nature..

[9] de Ligt J, Willemsen MH, van Bon BWM, Kleefstra T, Yntema HG, Kroes T (2012). Diagnostic exome sequencing in persons with severe intellectual disability. N Engl J Med.

[10] Lelieveld SH, Reijnders MRF, Pfundt R, Yntema HG, Kamsteeg E-J, de Vries P (2016). Meta-analysis of 2,104 trios provides support for 10 new genes for intellectual disability. Nat Neurosci.

[11] Rauch A, Wieczorek D, Graf E, Wieland T, Endele S, Schwarzmayr T (2012). Range of genetic mutations associated with severe non-syndromic sporadic intellectual disability: an exome sequencing study. Lancet.

[12] Appenzeller S, Balling R, Barisic N, Baulac S, Caglayan H, Craiu D (2014). De novo mutations in synaptic transmission genes including DNM1 cause epileptic encephalopathies. Am J Hum Genet.

[13] Heyne HO, Singh T, Stamberger H, Abou Jamra R, Caglayan H, Craiu D (2018). De novo variants in neurodevelopmental disorders with epilepsy. Nat Genet.

[14] Lek M, Karczewski KJ, Minikel EV, Samocha KE, Banks E, Fennell T (2016). Analysis of protein-coding genetic variation in 60,706 humans. Nature..

[15] Tan A, Abecasis GR, Kang HM (2015). Unified representation of genetic variants. Bioinformatics..

[16] Wang K, Li M, Hakonarson H (2010). ANNOVAR: functional annotation of genetic variants from high-throughput sequencing data. Nucleic Acids Res.

[17] Goode DL, Cooper GM, Schmutz J, Dickson M, Gonzales E, Tsai M (2010). Evolutionary constraint facilitates interpretation of genetic variation in resequenced human genomes. Genome Res.

[18] Vilella AJ, Severin J, Ureta-Vidal A, Heng L, Durbin R, Birney E (2008). EnsemblCompara GeneTrees: complete, duplication-aware phylogenetic trees in vertebrates. Genome Res.

[19] Farrell CM, O’Leary NA, Harte RA, Loveland JE, Wilming LG, Wallin C (2014). Current status and new features of the Consensus Coding Sequence database. Nucleic Acids Res.

[20] Kinsella RJ, Kähäri A, Haider S, Zamora J, Proctor G, Spudich G (2011). Ensembl BioMarts: a hub for data retrieval across taxonomic space. Database J Biol Databases Curation.

[21] Edgar RC (2004). MUSCLE: multiple sequence alignment with high accuracy and high throughput. Nucleic Acids Res.

[22] Chen S, Krinsky BH, Long M (2013). New genes as drivers of phenotypic evolution. Nat Rev Genet.

[23] Dufayard J-F, Duret L, Penel S, Gouy M, Rechenmann F, Perriere G (2005). Tree pattern matching in phylogenetic trees: automatic search for orthologs or paralogs in homologous gene sequence databases. Bioinformatics..

[24] Waterhouse AM, Procter JB, Martin DMA, Clamp M, Barton GJ (2009). Jalview version 2--a multiple sequence alignment editor and analysis workbench. Bioinformatics..

[25] Livingstone CD, Barton GJ (1993). Protein sequence alignments: a strategy for the hierarchical analysis of residue conservation. Bioinformatics..

[26] Rand WM (1971). Objective criteria for the evaluation of clustering methods. J Am Stat Ass.

[27] Samocha KE, Robinson EB, Sanders SJ, Stevens C, Sabo A, McGrath LM (2014). A framework for the interpretation of de novo mutation in human disease. Nat Genet.

[28] Lonsdale J, Thomas J, Salvatore M, Phillips R, Lo E, Shad S (2013). The Genotype-Tissue Expression (GTEx) project. Nat Genet.

[29] Kosmicki JA, Samocha KE, Howrigan DP, Sanders SJ, Slowikowski K, Lek M (2017). Refining the role of de novo protein-truncating variants in neurodevelopmental disorders by using population reference samples. Nat Genet.

[30] Walsh R, Peters NS, Cook SA, Ware JS (2014). Paralogue annotation identifies novel pathogenic variants in patients with Brugada syndrome and catecholaminergic polymorphic ventricular tachycardia. J Med Genet.

[31] Peterson ME, Chen F, Saven JG, Roos DS, Babbitt PC, Sali A (2009). Evolutionary constraints on structural similarity in orthologs and paralogs. Protein Sci.

[32] DeLuna A, Vetsigian K, Shoresh N, Hegreness M, Colón-González M, Chao S (2008). Exposing the fitness contribution of duplicated genes. Nat Genet.

[33] Ori A, Iskar M, Buczak K, Kastritis P, Parca L, Andrés-Pons A, et al. Spatiotemporal variation of mammalian protein complex stoichiometries. Genome Biol 2016;17(47). 10.1186/s13059-016-0912-5. 10.1186/s13059-016-0912-5 PMC4791834 26975353

[34] Bar-Shira O, Maor R, Chechik G (2015). Gene Expression switching of receptor subunits in human brain development. PLOS Comput Biol.

[35] Thompson PM, Gotoh T, Kok M, White PS, Brodeur GM (2003). CHD5, a new member of the chromodomain gene family, is preferentially expressed in the nervous system. Oncogene..

[36] Lal D, May P, Samocha K, Kosmicki J, Robinson EB, Moller R, et al. Gene family information facilitates variant interpretation and identification of disease-associated genes. bioRxiv 2017 159780. 10.1186/s13073-020-00725-6 PMC7079346 32183904

[37] Pérez-Palma E, May P, Iqbal S, Niestroj L-M, Du J, Heyne HO (2020). Identification of pathogenic variant enriched regions across genes and gene families. Genome Res.

[38] May P, Lal D. Paralog variant classification and scoring Zenodo. 2019. 10.5281/zenodo.3582386.39. Accessed 18 Dec 2019.

[39] May, P, Lal D. Paralogs. Gitlab. https://git-r3lab.uni.lu/genomeanalysis/paralogs. Accessed 2 Feb 2020.

