# Supplementary material for: Gene family information facilitates variant interpretation and identification of disease-associated genes in neurodevelopmental disorders
Source: Genome Med. 2020 Mar 17;12:28. doi: 10.1186/s13073-020-00725-6 (PMC7079346; doi:10.1186/s13073-020-00725-6)
Supplement: Supplementary file 1 — Additional file 1: Supplementary methods, figures, and table. [file 13073_2020_725_MOESM1_ESM.docx]

Supplemental Material for the article:

**Gene family information facilitates variant interpretation and identification of disease-associated genes in neurodevelopmental disorders**

**Content** **Page**

Supplemental Methods 1

Supplementary Figure S1: **Paralog conservation** 2

Supplementary Figure S2: **Paralog conservation workflow** 3

Supplementary Figure S3: **Correlation analysis of pathogenicity and conservation scores** 4

Supplementary Figure S4: **Screenshot for paralog conservation viewer** 5

Supplementary Table S1: **New significant paralog intolerant NDD candidate genes** 7

References 9

**Supplemental Methods**

**Comparison of para_zscore with other pathogenicity and conservation score**

We compared 5000 missense variants from our NDD cohort together with 5000 randomly extracted missense variants from ExAC (1) (with MAF< 0.05). For each variant we calculated the Spearman rank correlation between the following scores: the para_zscores for the original protein families (PARASCOREzSCORE), the para_zscores for protein families finally sub-clustered at 80% identity level (PARASUBSCOREzSCORE), which is the *para_zscore* that we used throughout the paper to define paralog conserved residue positions. From the dbNFSP (2) we extracted the scores for MutationTaster (3), GERP++ (4), SiPhy (5), MutationAssessor (6), VEST3 (7), CADD (8), PolyPhen-2 (9), FATHHMM (10), SIFT (11), LRT (12). The pairwise correlation matrix is given in Supplementary Figure 1. Correlations were computed with R v.3.4.1.

**
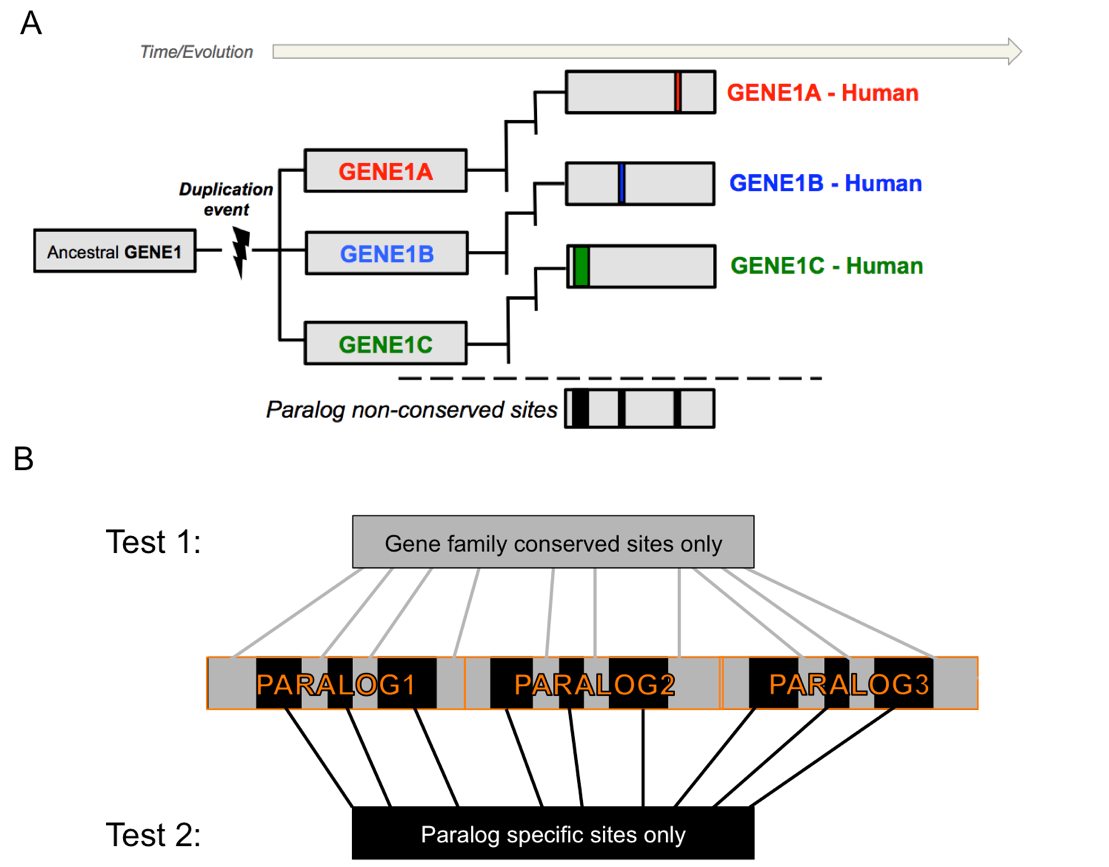
**

**Figure S1: Paralog conservation.** Schematic overview of paralog conservation (A) and the gene family enrichment tests of paralog conserved vs. non-conserved sites (B**).** Paralog conserved sites are summed over all members of the gene family. Paralog conserved sites are in black, paralog non-conserved sites in grey. Enrichment tests can be made for paralog conserved sites (Test 1) and paralog non-conserved sites (Test 2).

**
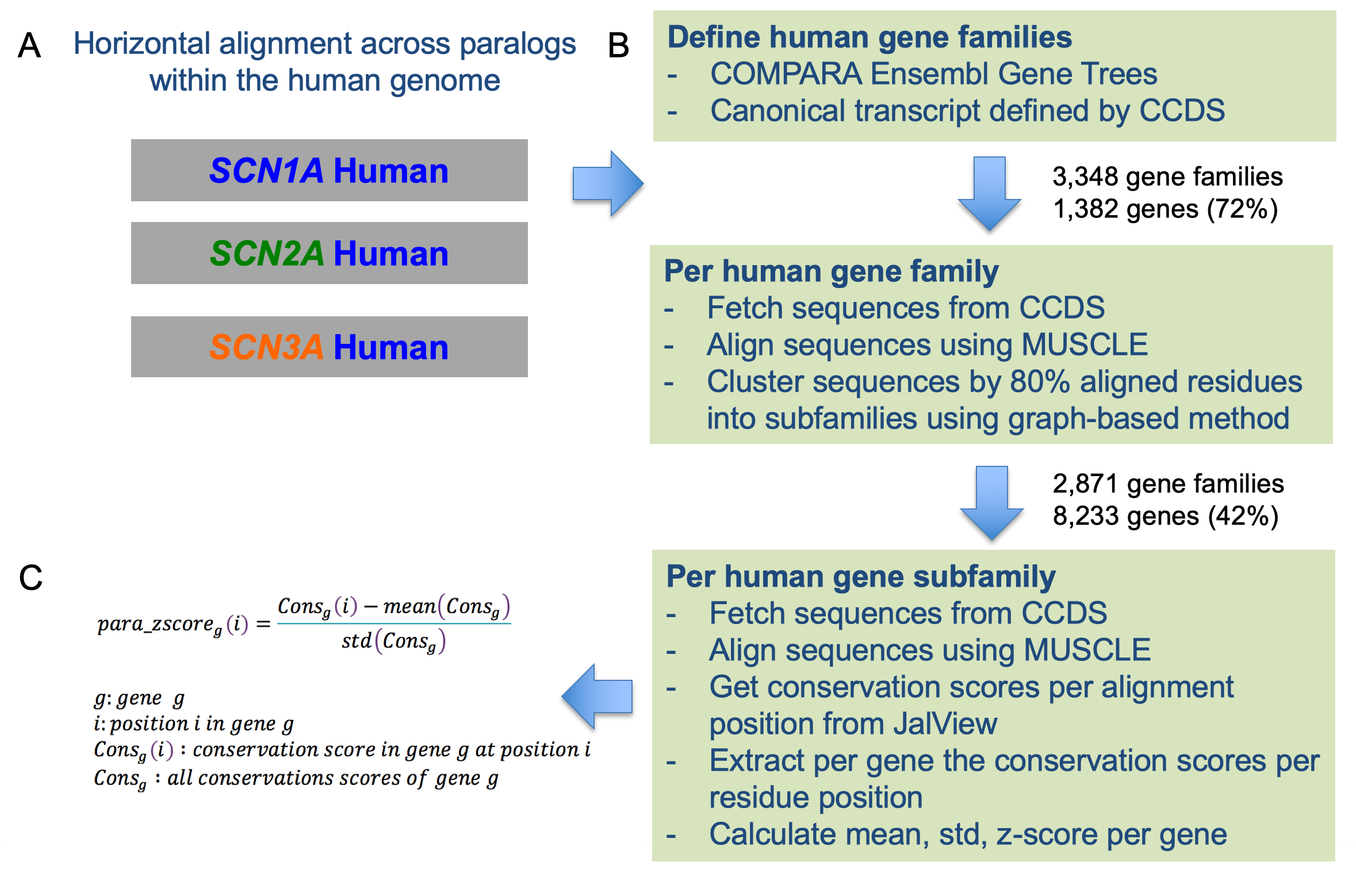
**

**Supplementary Figure S2: Paralog conservation workflow.** (A) Representation of paralog alignment for three members of the voltage-gated sodium channel gene family. (B) Workflow for the paralog conservation scoring using Ensembl family definitions and CCDS canonical transcript annotations as input. (C) Formula for para_zscore. Muscle (13), JalView (14).

**
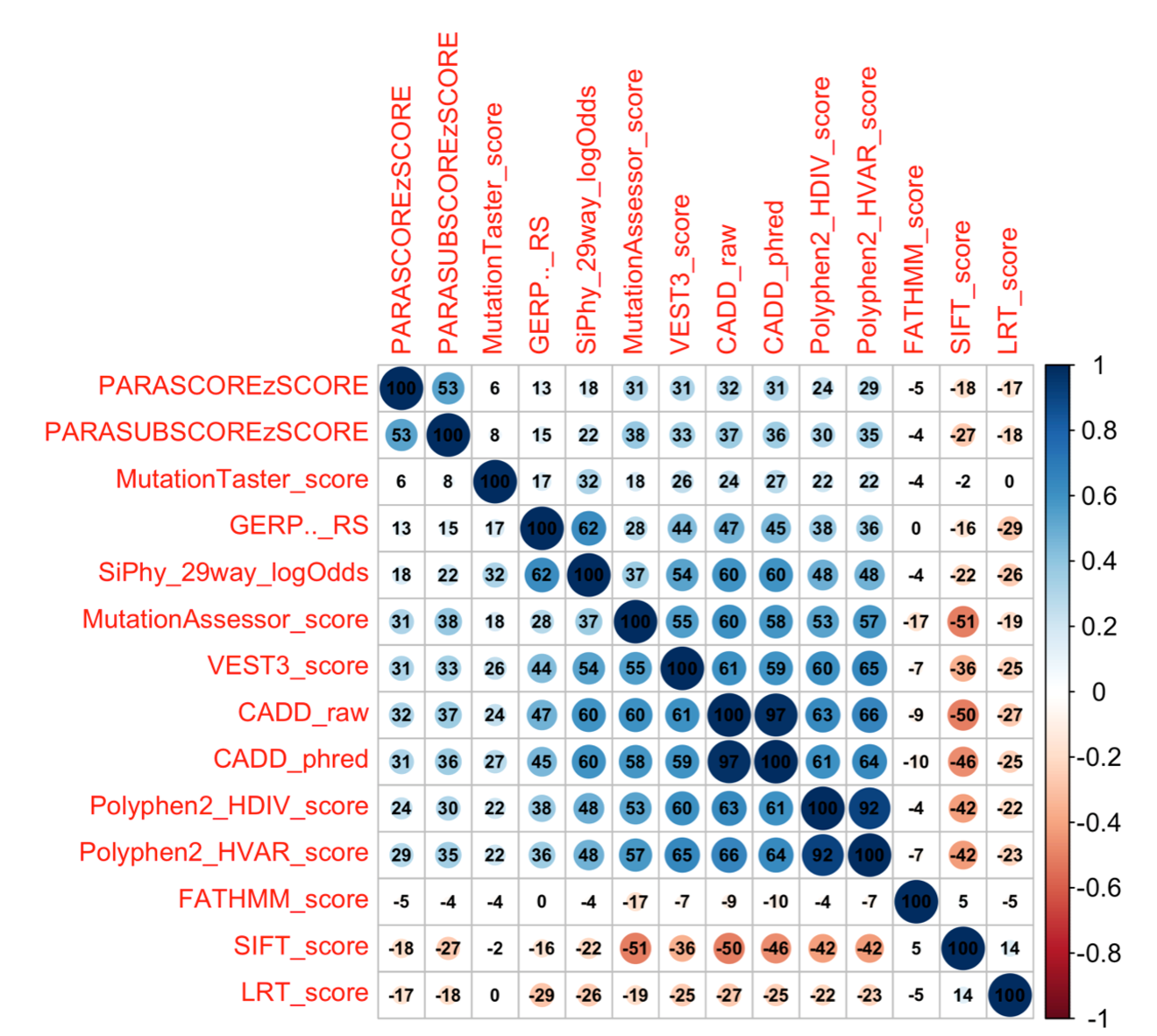
**

**Supplementary Figure S3: Correlation analysis of pathogenicity and conservation scores.** Pairwise Spearman rank correlation coefficients for randomly selected 5000 DNM and 5000 ExAC variants.


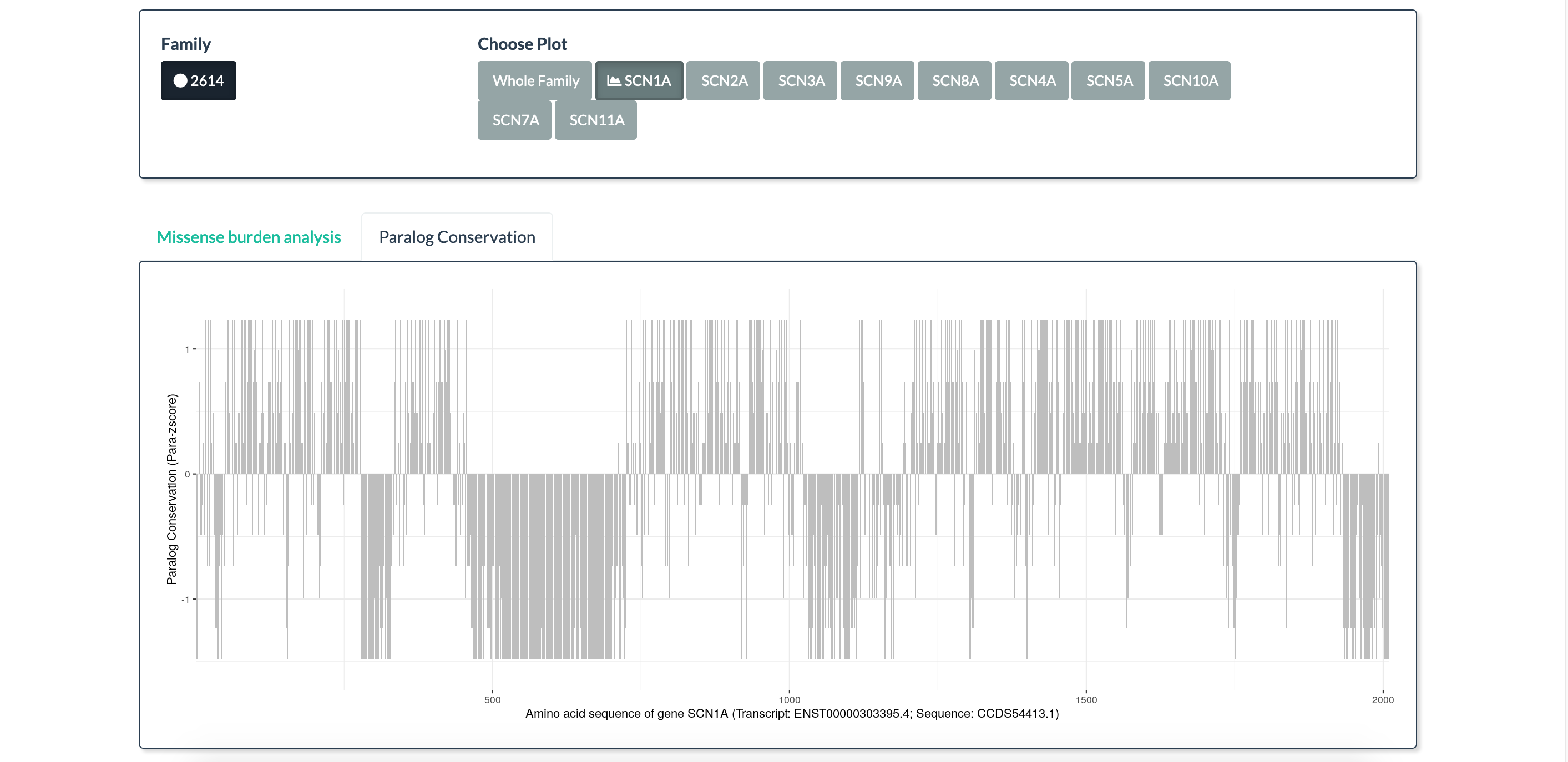


**Supplementary Figure S4: Screenshot for paralog conservation viewer.** Paralog conservation for SCN1A from the paralog family 2614 given as para_zscore over the entire amino acid sequence of SCN1A. Screenshot taken from http://per.broadinstitute.org/ (15).

| **Gene** | **N**  **DNM total** | **Mean Brain RPKM** | **mis_z** | **pLI** | **DD- DNM** | **ASD- DNM** | **EPI -DNM** | **Expression profile (GTEx)** | **Mouse neurological phenotype^16^** | | **Human**  **NDD phenotype** | **Functional support** | **Additional Comments** |
| --- | --- | --- | --- | --- | --- | --- | --- | --- | --- | --- | --- | --- | --- |
| *HECW2* | 9 | 1.68 | 3.49 | 1 | 7 | 1 | 1 | = | - | DD^17^, Hyp^17^, MR^3^ | | - | Mutations lethal in mice^4^, GWAS aging^20^. |
| *CACNA1A* | 9 | 25.9 | 7.25 | 1 | 7 | 0 | 2 | +++ | Yes | EPI^21^ | | *in vivo*^21^ | Known disease gene for spinocerebellar ataxia, familiar hemiplegic migraine^22^. Strong mouse model support. Heterozygous mice exhibits seizures^23^. |
| *CHD7* | 7 | 5.94 | 2.24 | 1 | 6 | 1 | 0 | +++ | Yes | DD^24^ | | *in vivo*^24^ | CHARGE syndrome. Essential for mouse brain development^25^, and mutations lead to abnormal nervous system development^26^. |
| *TCF7L2* | 6 | 3.69 | 3.21 | 1 | 2 | 4 | 0 | = | Yes | DD^27,28^ MR^29^ | |  | Pitt-Hopkins syndrome. DNMs in ASD patients from Iossifov et al, 2014^30^. |
| *GRIN2A* | 6 | 3.19 | 4.15 | 1 | 5 | 0 | 1 | +++ | Yes | ARE^31^,  EPI/APh^32^ | | *ex vivo*^31^,  *in vitro*^32^ | Mice show neurological and behavioral phenotype^32^. |
| *ARID1A* | 5 | 8.48 | 4.61 | 1 | 5 | 0 | 0 | = | Yes | DD^33^ | | - | Coffin-Siris syndrome. Heterozygous mice show developmental phenotype including altered nervous system^34^. |
| *CHD5* | 5 | 16.91 | 6.4 | 1 | 4 | 1 | 0 | = | Yes | DD^35^, MR^36^ | | - | Gene is located in the 1p36 deletion (a DD) syndrome loci^35^. |
| *SCN3A* | 4 | 4.36 | 4.73 | 1 | 1 | 3 | 0 | +++ | Yes^37^ | DD/ASD^38^, EPI^37,39-43^ | | *in vitro*^41^ | DNMs in ASD patients^44^ |
| *RAB11A* | 3 | 21.95 | 3.38 | 0.98 | 3 | 0 | 0 | = | - | - | | - | - |
| *TCF12* | 3 | 6.83 | 0.54 | 0.99 | 3 | 0 | 0 | = | Yes | CrS/DD/ ASD^45^ | | *in vivo*^45^ | - |
| *KCNQ5* | 3 | 6.1 | 4.64 | 1 | 3 | 0 | 0 | +++ | Yes^46^ | - | | - | Likely pathogenic mutations in patients with MR and ASD, absence of direct associations^46,47^. |
| *CHD1* | 3 | 3.68 | 3.25 | 1 | 1 | 2 | 0 | = | Yes | - | | - | The gene regulates pluripotency of embryonic stem cells, when lost cells have propensity to differentiate into neurons^48^. |
| *RAB2A* | 2 | 30.8 | 3.29 | 0.96 | 1 | 1 | 0 | = | - | ASD/SCZ^49^ | | - | LoF and synonymous mutations likely affecting splicing have been associated with ASD and SCZ^49^. |
| *RAB14* | 2 | 29.34 | 3.31 | 0.96 | 2 | 0 | 0 | = | - | - | | - | - |
| *GABRB1* | 2 | 1.5 | 3.54 | 0.96 | 0 | 1 | 1 | +++ | Yes | ASD^50^, EPI^51,52^ | | *in vitro*^52^ | bipolar disorder GWAS association lay within GABRB1^53^. |
| *NFIB* | 2 | 6.68 | 3.02 | 0.98 | 1 | 1 | 0 | = | Yes | ASD^54^ | | *in vitro*^54^ | Proposed as autism susceptibility gene^54^. |
| *HDAC3* | 2 | 17.56 | 4.39 | 0.91 | 2 | 0 | 0 | = | Yes | - | | - | HDAC3 plays an important role in transcriptional regulation, cell cycle progression and developmental events^55^. |
| *MEF2D* | 2 | 16.46 | 2.55 | 1 | 2 | 0 | 0 | = | Yes | - | | - | TF targets are mutated in EPI and ASD patients^56^. |
| *NFIA* | 2 | 7.54 | 3.2 | 1 | 1 | 1 | 0 | +++ | Yes | DD^57,58^; MaC/ HypP^59,60^ | | - | TF modulates oligodendrocytes and astrocytes differentiation^61^. |
| *MED13* | 2 | 4.9 | 1.7 | 1 | 0 | 2 | 0 | = | - | MR, Dys^62^ | | - | - |
| *GNA11* | 1 | 18.46 | 4.98 | 0.96 | 1 | 0 | 0 | = | Yes | HypP, MR, Dys^63^ | | - | - |
| *SOX9* | 1 | 18.72 | 4.11 | 0.98 | 1 | 0 | 0 | = | - | - | | - | TF modulates mouse astrocytes diferentiation^64^. Downregulated in ASD^65^. |
| *PPP2R5A* | 1 | 13.73 | 2.5 | 0.96 | 1 | 0 | 0 | = | - | - | | - | -. |
| *DLG2* | 1 | 12.64 | 1.85 | 0.91 | 0 | 1 | 0 | +++ | Yes | ASD (CNVs)^66^ | | - | DLG2 encodes the PSD protein known as chapsyn-110^66^. GWAS of ADHD^67^. |
| *CACNA1B* | 1 | 8.35 | 4.38 | 1 | 0 | 0 | 1 | +++ | Yes | MyoDys^68,69^ | | *in vitro*^69^ | - |
| *HECW1* | 1 | 1.65 | 3.55 | 1 | 1 | 0 | 0 | +++ | - | - | | - | - |
| *CHD9* | 1 | 3.39 | 2.64 | 1 | 1 | 0 | 0 | = | - | - | | - | - |
| *USP24* | 1 | 8.87 | 3.06 | 1 | 0 | 1 | 0 | +++ | - | - | | - | - |

**Table S1. New significant paralog intolerant NDD candidate genes.** 28 genes with de novo mutations that are expressed in brain and that show paralog intolerance for either missense and/or loss-of-function variation of gene families enriched for variants in NDD patients. None of the genes have previously been reported as significant in an exome-wide association study in NDD patients, even though some genes mentioned above have previously been associated with known neurological or neurodevelopmental disorders.

**References**

1. Lek M. *et al.* Analysis of protein-coding genetic variation in 60,706 humans. *Nature* **536,** 285-291 (2016).

2. Liu X. *et al.* dbNFSP v.3.0: A One-Stop Database of Functional Predictions and Annotations for Human Non-synonymous and Splice-Site SNVs. *Human Mutat* **37**, 235-241 (2016).

3. Schwarz J.M. *et al.* MutationTaster2: mutation prediction for the deep-sequencing age. *Nat Methods* **11**, 361-362 (2014).

4. Davydov E.V. *et al.* Identifying a high fraction of the human genome to be under selective constraint using GERP++. *PLoS Comput Biol* **6**, e1001025 (2010).

5. Garber M. *et al.* Identifying novel constraint elements by exploiting biased substitution patterns. *Bioinformatics* **25**, i54-62 (2009).

6. Reva B. *et al.* Predicting the Functional Impact of Protein Mutations: Application to Cancer Genomics. *Nucleic Acids Res* **39**, e118 (2011).

7. Carter H *et al.* Identifying Mendelian disease genes with the Variant Effect Scoring Tool. *BMC Genomics* **14**, 1-16 (2013).

8. Kircher M. *et al.* A general framework for estimating the relative pathogenicity of human genetic variants. *Nat Genet* **46**, 310-315 (2014).

9. Adzhubei I. *et al.* Predicting Functional Effect of Human Missense Mutations Using PolyPhen-2. *Curr Protoc Hum Genet*, Chapter 7 (2013).

10. Shihab H.A., *et al.* Predicting the Functional, Molecular and Phenotypic Consequences of Amino Acid Substitutions using Hidden Markov Models. *Hum Mutat* **34**, 57-65 (2013).

11. Kumar P. *et al.* Predicting the effects of coding non-synonymous variants on protein function using the SIFT algorithm. *Nat Protoc* **4**, 1073-1081 (2009).

12. Chun S. *et al.* Identification of deleterious mutations within three human genomes. *Genome Res* **19**, 1553-1561 (2009).

13. Edgar R.C. MUSCLE: multiple sequence alignment with high accuracy and high throughput. *Nucleic Acids Res* **32**. 1792–1797 (2004).

14 Waterhouse A.M. *et al.* Jalview Version 2--a multiple sequence alignment editor and analysis workbench. *Bioinformatics* **25**,1189–1191 (2009).

15 Pérez-Palma E. *et al.* [Identification of pathogenic variant enriched regions across genes and gene families](https://www.biorxiv.org/content/10.1101/641043v1). *bioRxiv* **641043** (2019).

16 Blake, J. A. *et al.* Mouse Genome Database (MGD)-2017: community knowledge resource for the laboratory mouse. *Nucleic acids research* **45**, D723-D729, (2017).

17 Berko, E. R. *et al.* De novo missense variants in HECW2 are associated with neurodevelopmental delay and hypotonia. *Journal of medical genetics* **54**, 84-86 (2017).

18 Halvardson, J. *et al.* Mutations in HECW2 are associated with intellectual disability and epilepsy. *Journal of medical genetics* **53**, 697-704 (2016).

19 Koscielny, G. *et al.* The International Mouse Phenotyping Consortium Web Portal, a unified point of access for knockout mice and related phenotyping data. *Nucleic acids research* **42**, D802-809 (2014).

20 Walter, S. *et al.* A genome-wide association study of aging. *Neurobiology of aging* **32**, 2109 e2115-2128 (2011).

21 Bomben, V. C. *et al.* Isolated P/Q Calcium Channel Deletion in Layer VI Corticothalamic Neurons Generates Absence Epilepsy. *The Journal of neuroscience : the official journal of the Society for Neuroscience* **36**, 405-418 (2016).

22 Dilekoz, E. *et al.* Migraine mutations impair hippocampal learning despite enhanced long-term potentiation. *The Journal of neuroscience : the official journal of the Society for Neuroscience* **35**, 3397-3402 (2015).

23 Kim, T. Y. *et al.* Absence-like seizures and their pharmacological profile in tottering-6j mice. *Biochemical and biophysical research communications* **463**, 148-153 (2015).

24 Micucci, J. A. *et al.* CHD7 and retinoic acid signaling cooperate to regulate neural stem cell and inner ear development in mouse models of CHARGE syndrome. *Human molecular genetics* **23**, 434-448 (2014).

25 Feng, W. *et al.* Chd7 is indispensable for mammalian brain development through activation of a neuronal differentiation programme. *Nature communications* **8**, 14758 (2017).

26 Jiang, X. *et al.* The mutation in Chd7 causes misexpression of Bmp4 and developmental defects in telencephalic midline. *The American journal of pathology* **181**, 626-641 (2012).

27 Giurgea, I. *et al.* TCF4 deletions in Pitt-Hopkins Syndrome. *Human mutation* **29**, E242-251 (2008).

28 Zweier, C. *et al.* Haploinsufficiency of TCF4 causes syndromal mental retardation with intermittent hyperventilation (Pitt-Hopkins syndrome). *American journal of human genetics* **80**, 994-1001 (2007).

29 Kalscheuer, V. M. *et al.* Disruption of the TCF4 gene in a girl with mental retardation but without the classical Pitt-Hopkins syndrome. *American journal of medical genetics. Part A* **146A**, 2053-2059 (2008).

30 Iossifov, I. *et al.* Low load for disruptive mutations in autism genes and their biased transmission. *Proc Natl Acad Sci U S A* **112**, E5600-

7(2015).

31 Lemke, J. R. *et al.* Mutations in GRIN2A cause idiopathic focal epilepsy with rolandic spikes. *Nature genetics* **45**, 1067-1072 (2013).

32 Carvill, G. L. *et al.* GRIN2A mutations cause epilepsy-aphasia spectrum disorders. *Nature genetics* **45**, 1073-1076 (2013).

33 Zarate, Y. A. *et al.* SMARCE1, a rare cause of Coffin-Siris Syndrome: Clinical description of three additional cases. *American journal of medical genetics. Part A* **170**, 1967-1973 (2016).

34 Chandler, R. L. & Magnuson, T. The SWI/SNF BAF-A complex is essential for neural crest development. *Developmental biology* **411**, 15-24 (2016).

35 Shimada, S. *et al.* Microarray analysis of 50 patients reveals the critical chromosomal regions responsible for 1p36 deletion syndrome-related complications. *Brain & development* **37**, 515-526 (2015).

36 Isidor, B. *et al.* Complex constitutional subtelomeric 1p36.3 deletion/duplication in a mentally retarded child with neonatal neuroblastoma. *European journal of medical genetics* **51**, 679-684 (2008).

37 Lamar, T. *et al.* SCN3A deficiency associated with increased seizure susceptibility. *Neurobiology of disease* **102**, 38-48 (2017).

38 Celle, M. E., Cuoco, C., Porta, S., Gimelli, G. & Tassano, E. Interstitial 2q24.3 deletion including SCN2A and SCN3A genes in a patient with autistic features, psychomotor delay, microcephaly and no history of seizures. *Gene* **532**, 294-296 (2013).

39 Heron, S. E. *et al.* Familial neonatal seizures with intellectual disability caused by a microduplication of chromosome 2q24.3. *Epilepsia* **51** (2010).

40 Bartnik, M., Chun-Hui Tsai, A., Xia, Z., Cheung, S. W. & Stankiewicz, P. Disruption of the SCN2A and SCN3A genes in a patient with mental retardation, neurobehavioral and psychiatric abnormalities, and a history of infantile seizures. *Clinical genetics* **80**, 191-195 (2011).

41 Vanoye, C. G., Gurnett, C. A., Holland, K. D., George, A. L., Jr. & Kearney, J. A. Novel SCN3A variants associated with focal epilepsy in children. *Neurobiology of disease* **62**, 313-322 (2014).

42 Chen, Y. J. *et al.* Electrophysiological Differences between the Same Pore Region Mutation in SCN1A and SCN3A. *Molecular neurobiology* **51**, 1263-1270 (2015).

43 Thuresson, A. C. *et al.* Whole gene duplication of SCN2A and SCN3A is associated with neonatal seizures and a normal intellectual development. *Clinical genetics* **91** (2017).

44 Turner, T. N. *et al.* Genomic Patterns of De Novo Mutation in Simplex Autism. *Cell* **171**, 710-722 e712 (2017).

45 Sharma, V. P. *et al.* Mutations in TCF12, encoding a basic helix-loop-helix partner of TWIST1, are a frequent cause of coronal craniosynostosis. *Nature genetics* **45** (2013).

46 Fidzinski, P. *et al.* KCNQ5 K(+) channels control hippocampal synaptic inhibition and fast network oscillations. *Nature communications* **6**, 6254 (2015).

47 Gilling, M. *et al.* Dysfunction of the Heteromeric KV7.3/KV7.5 Potassium Channel is Associated with Autism Spectrum Disorders. *Frontiers in genetics* **4**, 54 (2013).

48 Gaspar-Maia, A. *et al.* Chd1 regulates open chromatin and pluripotency of embryonic stem cells. *Nature* **460**, 863-868 (2009).

49 Takata, A., Ionita-Laza, I., Gogos, J. A., Xu, B. & Karayiorgou, M. De Novo Synonymous Mutations in Regulatory Elements Contribute to the Genetic Etiology of Autism and Schizophrenia. *Neuron* **89**, 940-947 (2016).

50 Srivastava, S. *et al.* A novel variant in GABRB2 associated with intellectual disability and epilepsy. *American journal of medical genetics. Part A* **164A**, 2914-2921 (2014).

51 Lien, E., Vatevik, A. K., Ostern, R., Haukanes, B. I. & Houge, G. A second patient with a De Novo GABRB1 mutation and epileptic encephalopathy. *Annals of neurology* **80**, 311-312 (2016).

52 Janve, V. S., Hernandez, C. C., Verdier, K. M., Hu, N. & Macdonald, R. L. Epileptic encephalopathy de novo GABRB mutations impair GABAA receptor function. *Annals of neurology* (2016).

53 Craddock, N. *et al.* Strong genetic evidence for a selective influence of GABAA receptors on a component of the bipolar disorder phenotype. *Molecular psychiatry* **15**, 146-153 (2010).

54 Choi, J., Ababon, M. R., Matteson, P. G. & Millonig, J. H. Cut-like homeobox 1 and nuclear factor I/B mediate ENGRAILED2 autism spectrum disorder-associated haplotype function. *Human molecular genetics* **21**, 1566-1580 (2012).

55 Wu, C. H. *et al.* The Universal Protein Resource (UniProt): an expanding universe of protein information. *Nucleic acids research* **34**, D187-191 (2006).

56 Flavell, S. W. *et al.* Genome-wide analysis of MEF2 transcriptional program reveals synaptic target genes and neuronal activity-dependent polyadenylation site selection. *Neuron* **60**, 1022-1038 (2008).

57 Coci, E. G. *et al.* CANPMR syndrome and chromosome 1p32-p31 deletion syndrome coexist in two related individuals affected by simultaneous haplo-insufficiency of CAMTA1 and NIFA genes. *Molecular cytogenetics* **9**, 10 (2016).

58 Mikhail, F. M. *et al.* Clinically relevant single gene or intragenic deletions encompassing critical neurodevelopmental genes in patients with developmental delay, mental retardation, and/or autism spectrum disorders. *American journal of medical genetics. Part A* **155A**, 2386-2396 (2011).

59 Koehler, U. *et al.* A novel 1p31.3p32.2 deletion involving the NFIA gene detected by array CGH in a patient with macrocephaly and hypoplasia of the corpus callosum. *European journal of pediatrics* **169**, 463-468 (2010).

60 Rao, A. *et al.* An intragenic deletion of the NFIA gene in a patient with a hypoplastic corpus callosum, craniofacial abnormalities and urinary tract defects. *European journal of medical genetics* **57**, 65-70 (2014).

61 Glasgow, S. M. *et al.* Mutual antagonism between Sox10 and NFIA regulates diversification of glial lineages and glioma subtypes. *Nature neuroscience* **17**, 1322-1329 (2014).

62 Boutry-Kryza, N. *et al.* An 800 kb deletion at 17q23.2 including the MED13 (THRAP1) gene, revealed by aCGH in a patient with a SMC 17p. *American journal of medical genetics. Part A* **158A**, 400-405 (2012).

63 Al-Kateb, H. *et al.* Molecular characterization of a novel, de novo, cryptic interstitial deletion on 19p13.3 in a child with a cutis aplasia and multiple congenital anomalies. *American journal of medical genetics. Part A* **152A**, 3148-3153 (2010).

64 Matsui, K. & Arai, T. Protective immunity induced by porin in experimental mouse salmonellosis. *Microbiology and immunology* **33**, 699-708 (1989).

65 Ghahramani Seno, M. M. *et al.* Gene and miRNA expression profiles in autism spectrum disorders. *Brain research* **1380**, 85-97, (2011).

66 Egger, G. *et al.* Identification of risk genes for autism spectrum disorder through copy number variation analysis in Austrian families. *Neurogenetics* **15**, 117-127 (2014).

67 Alemany, S. *et al.* New suggestive genetic loci and biological pathways for attention function in adult attention-deficit/hyperactivity disorder. *American journal of medical genetics. Part B, Neuropsychiatric genetics : the official publication of the International Society of Psychiatric Genetics* **168**, 459-470 (2015).

68 Mencacci, N. E. *et al.* The CACNA1B R1389H variant is not associated with myoclonus-dystonia in a large European multicentric cohort. *Human molecular genetics* **24**, 5326-5329 (2015).

69 Groen, J. L. *et al.* CACNA1B mutation is linked to unique myoclonus-dystonia syndrome. *Human molecular genetics* **24**, 987-993 (2015).
